# Person-attuned musical interactions (PAMI) in dementia care. Complex intervention research for constructing a training manual

**DOI:** 10.3389/fmed.2023.1160588

**Published:** 2023-05-02

**Authors:** Hanne Mette Ridder, Julie Kolbe Krøier, Jens Anderson-Ingstrup, Orii McDermott

**Affiliations:** ^1^Centre for Documentation and Research in Music Therapy, Department of Communication and Psychology, Aalborg University, Aalborg, Denmark; ^2^Faculty of Medicine and Health Sciences, University of Nottingham, Nottingham, United Kingdom

**Keywords:** residential care home, dementia, person-centered care, music therapy, attunement, complex interventions

## Abstract

**Introduction:**

Music is of vital importance for cognition, human care, and the formation of social communities throughout life. Dementia is a neurocognitive disorder that affects cognitive domains, and in late-stage dementia, care is needed in all aspects of daily living. Within residential care home contexts, carers play a significant role for the “caring culture” but often lack professional training in verbal and non-verbal communication skills. Thus, there is a need for training carers to respond to the multidimensional needs of persons with dementia. Music therapists use musical interactions but are not trained to train carers. Therefore, our aim was to explore person-attuned musical interactions (PAMI), and additionally, to develop and evaluate a training manual to be used by music therapists when supporting and training carers in non-verbal communication with persons with late-stage dementia in residential care home contexts.

**Research process:**

With a realist perspective and systems thinking and within the framework for complex intervention research, the research group integrated several overlapping subprojects by applying a non-linear and iterative research process. Core elements related to person-centered dementia care as well as learning objectives were considered through the following four phases; Developing, Feasibility, Evaluation, and Implementation.

**Results:**

The result was a training manual for qualified music therapists to use when teaching and collaborating with carers about how to implement PAMI in dementia care. The manual included comprehensive resources, a clear structure for training, defined learning objectives, and integration of theory.

**Discussion:**

With increased knowledge about caring values and non-verbal communication, residential care home cultures may develop carer competencies and provide professional attuned care for persons with dementia. Further piloting and testing to examine the general effect on caring cultures is needed.

## Introduction

It is lunch time at the care home, and residents gather at the tables. Anna already sits in her chair. Her hands scrub off imaginary stains, with her movements becoming faster and faster, and the tone of her voice becomes even more shrill. Lis goes to Anna and sits down next to her. She is aware of her own breathing, takes a deep breath, and shortly closes her eyes. Anna's breathing is rapid and shallow. Lis takes another deep breath, then put her hand next to Anna's on the table. Anna touches her hand. Lis turns up her palm, and Anna takes her hand. Then Lis quietly hums Frere Jacque, slowly, with long phrases and with an airy voice. Anna keeps holding her hand, then looks at her. They have eye contact and Lis smiles. Today Anna manages to eat her lunch all on her own, without help—and without shouting. [Composite case example based on (1)].

In this description of working with a person with dementia (Anna), the professional carer (Lis) uses several non-verbal “techniques” and succeeds in making an often chaotic situation safe and calm. Not only for Anna, but also for the other residents. Lis has worked together with a music therapist and seen how the music therapist was able to calm down residents at a late stage of dementia. Lis copies what the music therapist does, but afterwards it is very difficult to tell her colleagues what she did. Mostly they find that Anna disturbs and believe that she should not sit together with the other residents at lunch time.

When Lis interacts with Anna in a slow tempo, inviting gestures and a gentle tone of voice, she uses non-verbal communication. Guidelines for dementia care recommend the use of positive body language and describe non-verbal communication as crucial. For example, the Alzheimer Society (2) explains that “As the disease advances, the person with Alzheimer's may rely on non-verbal communication, such as facial expressions or vocal sounds.”

In a scoping review on communication between nurses and older adults, non-verbal communication is described as an integral part of the nurse-patient relationship (3). The review showed that nurses most frequently use space and distance, touch, body movement, and aspects of the voice, when communicating with older adults. The researchers conclude that nurses should be self-aware of their non-verbal communication behaviors and should identify their own style of non-verbal communication and understand how to modify their interactions. In the review, music, singing, or humming were not included as a means of communication, however *vocalics* were described. Vocalics were associated with “elderspeak” including oversimplifying the language, speaking at a slow rate, loud, and with a demeaning tone, or speaking too fast or too loudly or in a too soft tone (3). In elderspeak, similar vocal cues as in infant-directed speech are used; shorter sentences, slower speech, and higher pitch (4). Both elderspeak and infant-directed speech are described by concepts such a pitch, tempo, timbre, tone, and dynamics, and according to a study by Hilton et al. (5), there are acoustic regularities in infant-directed vocalizations across cultures, and they consist of speech and song simultaneously. However, unlike infant-directed speech, elderspeak arises from implicit ageist stereotypes and carries goals of patronizing care (6). Research suggests that elderspeak negatively impacts the person in contrast to the positive impact of infant-directed speech on infants (7). Using familiar, preferred music, as Lis did in the example, can be a way to utilize the beneficial elements of infant-directed speech (such as pitch, tempo, timbre, tone, and dynamics), without adopting a patronizing tone. Below we will explore the links between the science of music and communication styles, in order to explain how using music can support non-verbal communication.

### Music and non-verbal communication

There are numerous definitions of what music is. We find the definition by the Dictionary.com (8) relevant: music is “an art of sound in time that expresses ideas and emotions in significant forms through the elements of rhythm, melody, harmony, and color”. Music in the form of songs shape a unified whole with many elements of predictability. Rhythm and tempo in a song creates a form or a gestalt in the way stanzas, lines and phrasing are shaped, which in turn affects our breathing. Therefore, in regard to rhythm and structure, music and speech have much in common, also when it comes to tonality. Music and speech are explained as two parallel systems in human development, with a system for language and one for music, and with the same basic mechanisms (9).

The early characteristics of communication in human development is by Mithen explained as holistic, multi-modal, manipulative, and musical. As infant-directed speech is described as a mixture of music and speech, musical terms such as intonation, rhythm and phrasing become useful designations of so-called non-verbal communication. Unique aspects of musical communication and intrinsic musicality are seen across cultures, and intrinsic musicality is therefore considered an innate competence. Such communicative musicality is crucial for the child's further cognitive development (10), and even with advanced Alzheimer's disease, musical memory is surprisingly well preserved (11). In this perspective, music is of vital importance for human care, attachment, and language development, but also for the coordination of movement (e.g., gait function, dance, and physical, repetitive work), the passing on of cultural knowledge, and the formation of social communities. The importance of music for cognitive development applies throughout life, also as a means to prevent dementia (12).

### Dementia and the residential care home context

Dementia is a global health challenge, but although dementia is the 7th leading cause of death, dementia research accounts for < 1.5% of total health research output (13). While the world is searching for a cure against dementia, the WHO states that research on dementia care has shifted from a focus on cognition and behavior changes to study quality of life, positive living with dementia, community participation and social and emotional communication [(13), p. 40]. Dementia is an umbrella term for various neurocognitive disorders and affects the following cognitive domains; attention, planning (and working memory), learning (and long-term memory), language, perception, and social cognition (14).

Persons with late-stage dementia need care in all aspects of daily living which is often an impossible task for spouses and relatives, therefore residential care is required. In care homes, qualified nurses provide medical care, and trained staff provide personal care and support. The majority of residents in care homes have dementia, but are undiagnosed (15). Globally, the true prevalence of dementia is underestimated which is likely to lead to inadequate planning for health and care services and has strong implications for public policy (16). When care home staff are educated and trained in person-centered care practices and work in a person-centered care (PCC) environment, job satisfaction improves, and both job-related stress and employee turnover is reduced (17). According to Rajamohan et al. (17), the culture shift toward PCC was first initiated in the United Kingdom and is a promising global innovation and important for staff for improving the quality of care delivered to care home residents. With PCC the person is central, rather than the disease, and carers integrate a number of defined positive interactions (18, 19).

When staff describe what motivates them to meaningfully engage with residents with late-stage dementia, they refer to a caring culture. A caring culture is an environment which promotes relational working and staff well-being, which is carried out with positive attitudes toward dementia and expresses caring values and the ambience of the environment. A caring culture also prioritizes the relationships between residents and staff, teamwork, and input from managers and supervisors (20). Based on semi-structured interviews with 21 staff members from seven nursing homes, Haunch et al. conclude that effective leadership and teamwork is crucial for facilitating staff to understand “their role to connect, understand, accept and empathize with residents, understand the importance of getting to know residents and express their own caring attributes” [(20), p. 11]. Nurses play a significant role for the caring culture, yet a systematic review reveals that nurses lack knowledge about dementia and PCC, verbal and non-verbal communication skills, and strategies to manage residents' coexisting behavioral and mental health problems. Thus, when training care home staff, Evripidou et al. (21) point at the need to include nurses in the training in order to respond to residents' multidimensional needs and be aware of new approaches to care and management.

### Effect of music therapy on dementia symptoms

In late-stage dementia, non-verbal communication may be overlooked or underestimated by busy care staff, and to prevent this, Clare and colleagues recommend music interventions as they offer fundamental, emotion-based opportunities for connecting with others (22). The positive effect of music interventions and music therapy in dementia care is documented in a number of meta-reviews and meta-analyses and show reduction in behavioral and emotional dementia symptoms (23), in depression (24, 25), in depression, anxiety and apathy (26), in aggression and agitation (27), and in reduction of anxiety and agitation along with increases in cognition and quality of life (28–30).

In a single case study on music therapeutic caregiving, Hammar et al. (31) explored caregiver singing and found that singing during morning care of persons with dementia may decrease negative expressed emotions and increase positive emotions. In a cross-sectional study with 285 nurses, Sung et al. found that most nursing staff held positive attitudes toward the use of music for people with dementia in long-term care facilities, but only a third had used music in practice and reported that they lacked resources and time to implement music therapy in practice (32). Sung et al. refer to Munro and Mount (33), when they define music therapy as the “controlled use of music and its influence on the human being to aid in physiological, psychological and emotional integration of individual during treatment of an illness or disability” [(32), p. 1777]. In this study, we define music therapy as “the clinical and evidence-based use of music interventions to accomplish individualized goals within a therapeutic relationship by a credentialed professional […]” (34). As we do not expect staff to train in music therapy, we suggest that staff are trained in how to carry out *musical interactions* within in a person-centered perspective.

A phenomenological study by Melhuish et al. (35) showed that the relationships between care home staff and residents with dementia were improved when staff took part in music therapy and dance-movement therapy for residents, and Isaac et al. (36) found that person-centered music intervention training workshops led to a decline in behavioral and psychological symptoms of dementia and a reduction in staff stress.

The music therapist Beer developed a 1-h training for caregivers and offered suggestions to guide music therapists in educating other professionals in the care of persons with late-stage dementia (37). In a study by Hsu et al. (38) of individual music therapy for managing neuropsychiatric symptoms in care home residents with dementia, it was additionally explored whether the intervention could serve as an ongoing training of carers. After each music therapy session, carers were presented with two selected video clips. The clips aimed to address how neuropsychiatric symptoms were minimized, and how the music therapist enhanced the residents' expressions, mood, and cognitive and sensorimotor functioning. After the programme, carers reported enhanced caregiving techniques which point at a promising learning outcome from the music therapist-carer communication. Further, Ray and Götell (39) found improved well-being and significant decreases in depression symptoms in nursing home residents with dementia after an intervention that combined music therapy and the training of nursing assistants to lead music activities. Their study showed that music therapy skills can be shared to extend music therapeutical benefits when a music therapist first achieves primary goals and then transfers instruction and facilitation to caregivers.

### Aim: manualising a training intervention

The culture shift toward PCC is important for carers^1^ for improving the quality of care delivered to care home residents. A caring culture prioritizes the relationships between residents and carers, teamwork, and input from managers and supervisors. Concepts drawn from the science of music may assist carers in understanding their non-verbal communication and communication styles in the care of residents with late-stage dementia. Unique aspects of musical communication and intrinsic musicality are innate competencies and of vital importance for the formation of social communities, attachment and care, and form an underlying theoretical understanding of music therapy. Further, as mentioned above, there is strong evidence for music therapy to reduce behavioral and psychological symptoms of dementia (23, 24, 26, 27).

With this study, we therefore wanted to explore how music therapists in collaboration with carers can support the use of non-verbal communication in dementia care. As non-verbal communication is a broad concept, our focus is on *musical interactions*. Interactions are reciprocal communicative actions by means of non-verbal information, such as body language, gestures, facial expressions, rhythm or sound [(40), p. 346]. In order to emphasize our theoretical understanding of dementia care, these musical interactions are *person-centered* according to PCC. However, we understand interactions as reciprocal, and that they should be attuned to the person with dementia, which we therefore term *person-attuned*.

Music therapists are trained to apply musical interactions, but they are not trained to train carers. To support them in this endeavor, we find it important to integrate aspects of transformative learning (41) in the training of carers, for example by not only relying on teaching theory, but by combining the training with action learning sessions and counseling in groups as well as directly at the workplace. In addition, we emphasize that, although already qualified, the music therapists should be trained to train.

In summing up, our aim is both to explore *person-attuned musical interactions* (PAMI), and further to develop and evaluate a training manual to be used by music therapists when supporting and training carers in non-verbal communication with persons with dementia in a residential care home context. The overall scope of the study is to stimulate a culture shift in caring cultures where carers are supported in a meaningful way to improve non-verbal communication through PAMI.

## Research process

### Researching complex interventions

In this study we address interactions carried out in daily practice from a specific theoretical perspective (PCC), we explore the meaning of these interactions and their context with the aim to distill knowledge to an overall theoretical perspective for music therapists to use when they collaborate with and share knowledge with carers. By exploring perspectives from the literature, as well as our own perspectives from practice, we intend to systematize knowledge into a concrete training manual and to test if it works and how it works.

According to the UK Health Technology Assessment 2021 (42), interventions are actions taken to make a change. Interventions are complex when we are dealing with several intervention components, when a range of behaviors, expertise and communication skills are required in complex settings, and when the interactions between interventions are dynamic and adaptive (43).

In the framework for the development and evaluation of complex interventions, the research process is divided into four phases: development, feasibility, evaluation, and implementation and includes both the content of the intervention and the context in which it is conducted (42). By including real world residential care home contexts and perspectives from carers and clinicians, we aim to emphasize interventions that are co-developed by carers and to include systems thinking methods (44). With a theory-based perspective, we seek to adopt PCC and music therapy theory and explore these perspectives in the context of residential dementia care, and with a systems perspective to explore how PAMI are interventions already existing in the system as tacit knowledge and caring values but suppressed by structural factors in the care home system. We expect that permitting PAMI as a part of professional care in some cases will disrupt rigid and dehumanized caring systems and lead to positive changes. Although our evaluation perspective regards systems, we set out with a realist perspective in order to answer what works in which circumstances and for whom (45).

**Figure 1:**
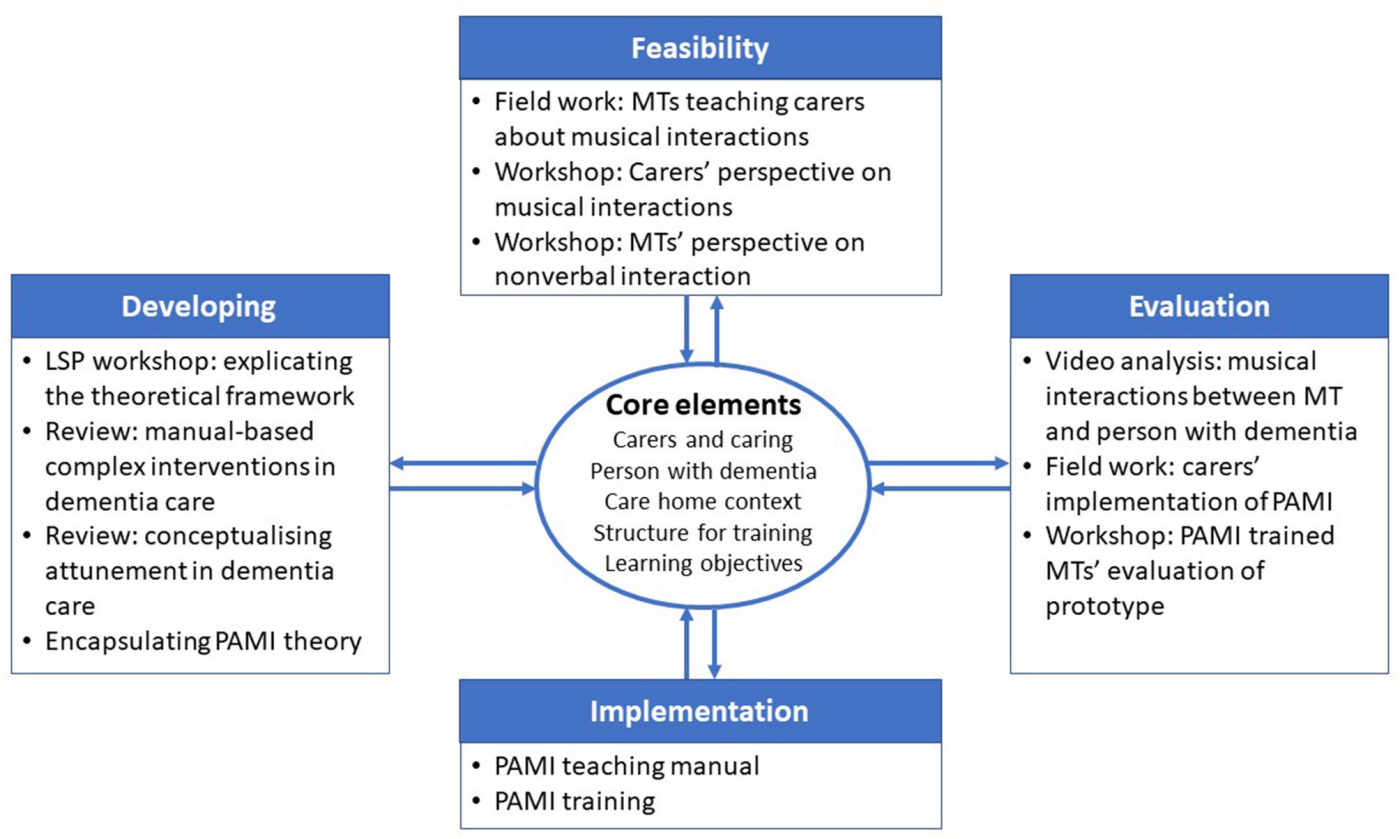
The iterative pathway through the phases of the PAMI research process based on the framework for complex intervention research by Skivington et al. [(42), p. 26].

Following the main phases and core elements of complex intervention research [(42), p. 26], we apply non-linear and iterative pathways through the research process. In Figure 1 we give an overview of the subprojects in the process of development, feasibility, evaluation, and implementation. In our reporting of the research, we are led by the SQUIRE 2.0 guidelines (46) that provide us with a framework for reporting new knowledge about how to improve healthcare and to describe system level work.

### Collaborative human science research

The Velux foundation is a philanthropic foundation that among others supports research in gerontology and cultural, social, and environmental projects. With a so-called core-group programme, the foundation funds research in the humanities and allied social science disciplines with the aim to advance collective projects. In 2014 our group applied for the PAMI-project, we were granted funding in 2015, and the research project finally started in 2016 and ran until April 2022. With additional funding from the Danish Alzheimer Society, the project included a team of a PI, a postdoc, two PhD fellows and a 1-year research position. The original 4-years research period was extended due to maternity and paternity leaves and the lock-down period due to the COVID-19 pandemic. Each researcher in the team integrated their individual research projects in the core group project, with all projects aiming at exploring PAMI and developing a training manual for music therapists to use in the training of carers.

### Ethics

The study was granted exemption from the Danish regional ethics committee (Den Videnskabsetiske Komité for Region Nordjylland, January 25, 2016, project N-20160002) and was registered at The Danish Data Protection Agency through Aalborg University. The study adheres to the Danish Code of Conduct for Research Integrity, Ministry of Higher Education and Science, 2014, and participants signed an agreement on the terms of participating in the study based on written and oral information provided by the researchers.

### Overview of the iterative research process

With non-linear and iterative pathways through the research process and with overlapping projects, we illustrate the 12 most important subprojects leading to the PAMI-manual guiding music therapists (MTs) to teach carers. Figure 1 is based on the framework for complex intervention research (42) and illustrates how core elements are considered in any phase of the process. In the following we describe each of the four phases (Developing, Feasibility, Evaluation, and Implementation), and the 12 associated subprojects.

### Developing

To develop something is an act of advancing or elaborating. Our aim was to develop a new intervention, however neither music therapy methods nor music therapists' training of carers is new. Several music therapists train carers as part of their work, either formally with planned courses or workshops, or informally when they collaborate with carers about daily practice. This part of the process was therefore also about identifying examples of interactions of relevance for PAMI theory, practice and training with the common goal to develop a manual that integrates theory, practice and research. In the following, we will provide a brief overview of four subprojects that informed the development of the PAMI manual.

#### Subproject 1. LSP workshop: explicating the theoretical framework

The objective of this subproject was to explore, articulate and conceptualize professional caregiving from a person-centered perspective and to identify essential components and their implications of positive person work between carer and person with late-stage dementia. Based on a hermeneutic constructivistic approach we used Lego Serious Play™ (LSP) as a method for exploring, articulating, and conceptualizing caregiver interactions. Five panelists with expertise in dementia care and/or musical interactions participated. The result was a co-created, condensed model suggesting an ideographic understanding of person-attuned interactions in caregiving, emphasizing knowledge about personhood and reciprocity, and the development of caregiver interactions skills. The conclusion was that caregivers represent an essential value in dementia care by introspection and mentalisation and by providing a feeling of safety through person-attuned interactions. Caregiver competencies depend on resources, culture, and interdisciplinary collaboration, which puts a strong demand on continuous training and supervision, and for political and societal priorities (47).

#### Subproject 2. Review: manual-based complex interventions in dementia care

A scoping review was carried out to investigate how manuals published in refereed journals explain procedures of complex interventions in dementia care, how they are structured, and their content disseminated. Nine manuals were identified and analyzed using a template analysis. The manuals allowed for tailored actions, and two-thirds showed a medium degree of flexibility. The types of dissemination elements varied, but all used written text, and none used illustrations or audio/video material. Based on this, recommendations for future manuals for complex interventions where non-verbal elements are important were provided, suggesting the inclusion of illustrations and audio/video material to describe actions, and for ways to tailor, structure, describe, and disseminate (48).

#### Subproject 3. Review: conceptualizing attunement in dementia care

Based on a meta-ethnographic review, findings from six dementia care studies applying the term attunement were synthesized. This showed that attunement in dementia care is understood as a dynamic process between carers and persons with dementia. Attunement is linked to the person-centered approach to care and understood as an emotional phenomenon where carers relate to the needs and emotions of the person with dementia. In addition, adjustments in tempo and timing within the interaction between carers and person with dementia are described as essential for attunement to happen (49).

#### Subproject 4. Encapsulating PAMI theory

As one of the last subprojects to be finalized, the theoretical understanding of PAMI was explained in book format. It started as a chapter in the training manual, however it was necessary to expand and elaborate, and the chapter became increasingly comprehensive. A Danish publisher was interested in publishing this theoretical content as a book for music therapists and educators, but also for informal and professional caregivers. In the first chapter of the book an introduction is given to non-verbal communication, reciprocal musical interaction, dementia, and loss of cognitive functioning. In chapter two each part of the acronym PAMI is explained: Person(hood), attunement, music(al) and interaction, and chapter three elaborates on the function of music in three parts, covering how music in very different ways is important for framing a setting, regulating arousal, and for reciprocal relationship. Chapter four gives examples from practice by presenting lived experience descriptions and ethical considerations, and chapter five focuses on transformative learning theory and the practical use of music care plans (1).

### Feasibility

In this phase of the research, the feasibility and acceptability of PAMI was explored and evaluated to prepare for the evaluation phase. We therefore consulted with music therapists and carers and carried out field work, workshops, and interviews. At this stage of developing a PAMI manual, we did not carry out an actual feasibility study but considered preparatory work to qualitatively explore real world implications and uncertainties.

#### Subproject 5. Field work: MTs teaching carers about musical interactions

Each of the research team members shared and integrated their own experiences from teaching carers and other music therapists. This included contexts from Denmark, the Faroe Islands, and Norway. In an ongoing, collaborative process, the group integrated their experiences and constructed teaching material (e.g. PowerPoint presentations) that was used, reused, and refined each time someone from the group used the material for teaching during the project period. This practical starting point revealed which theoretical understandings were necessary to include and expand upon. Apart from the groups' own teaching material, we interviewed music therapist Niseema Munk Madsen, who had developed a specific training programme, the Music Ambassadors model. With this programme, carers took part in five training sessions over 8 months (all together 16 h) where they were taught how to integrate music in daily care aiming to increase quality of life. The music therapist/course facilitator emphasized the active engagement of carers in the training, that the aim and structure of the training should be clearly negotiated with care home managers, and finally to take into consideration the previous training of the carers. In this context, the facilitator observed that the majority of the carers had limited previous training which did put demands to how to introduce theory (50).

#### Subproject 6. Workshop: carers' use of musical interactions

In a 4-h workshop with 11 carers and a music therapist, we introduced participants to a number of action learning exercises. One exercise featured carers to role-play in a dyad where carer A was required to guide carer B (who was in the role of a person with dementia) to rise from a chair and walk along with A. Carer A was instructed not to use verbal language, and to be either empathic or to be in a hurry. This very simple exercise was discussed vividly in the subsequent focus groups as for some carers, it was a highly thought-provoking experience to be guided non-verbally, and they reflected on how difficult it was to describe what worked and what did not. The workshops and the three focus group discussions were recorded, transcribed, and analyzed. Several carers used music in caregiving and explained the importance of feedback from the local music therapist, as it helped them to become conscious about their actions, and how actions could be named as a technique. For example, carers felt it helpful to learn how to down-regulate arousal to help someone relax (51).

#### Subproject 7. Workshop: music therapists' perspective on non-verbal interaction

With the aim to understand how music therapists use musical interactions in dementia care, explorative workshops were conducted to study such interactions through the music therapists' lived experience descriptions. The workshops included focus group interviews, and transcripts from these were analyzed by using a phenomenological approach. The findings were then further elaborated and peer validated through musical improvisation as an arts-based analytic approach. The results suggested that music therapists are guided by the vitality of persons with dementia, are aware of their own reactions, and sense the needs of the other through disciplined subjectivity and by attuning to non-verbal musical parameters (e.g., tempo, pitch and volume). The five overall themes from the analysis were: vitality, disciplined subjectivity, attunement, therapeutic presence, and validation (52).

### Evaluation

Evaluation in research is used to obtain unbiased estimates of effectiveness, however, it is equally important to assess the usefulness of information for decision-making. Our ambition with this study was to explore and explain PAMI and to provide clear guidelines and contextualized understandings about how to interact non-verbally in care home contexts. We evaluated how music therapists carry out PAMI in clinical settings, how carers implement PAMI, and how music therapists, who were trained according to a prototype of the PAMI-manual, evaluated the content and structure of the PAMI training programme.

#### Subproject 8. Video analysis: musical interactions between music therapist and person with dementia

With video data from a music therapy session, a detailed sequential analysis of music therapy interactions was carried out following principles from conversation analysis, including a phenomenological transcription of the video and extraction of data concerning musical parameters. The results showed how different types of tempo variations are applied in the process of connecting with a person with late-stage dementia. The process was described as a person-attuned musical arousal regulation process (PAMAR), leading to person-attuned musical interactions where the person with dementia and the music therapist interacted reciprocally and with greater equality regarding the initiation of their interactions (53).

#### Subproject 9. Field work: carer's implementation of PAMI

For 4 months a music therapy researcher visited a care home weekly and worked together with six carers to explore how they used and understood musical interactions in dementia care. By interpreting narratives with thick descriptions of musical interactions and transcripts from a series of workshops based on a draft PAMI manual, an understanding of musical interaction incorporating the perspective of the six participating carers was constructed. The findings illuminated how musical interactions create vitality, communication, and connectedness through attunement. In addition, the musical interactions served as a soundtrack of the life story of the person with dementia and could transform anxiety into reassurance. It was concluded that musical interactions such as listening to music, dancing, singing, playing instruments, and paying attention to musical parameters such as tempo and timing of movements, may provide carers with new approaches to meet the psychosocial needs of persons with dementia (54).

#### Subproject 10. Workshop: PAMI trained MTs' evaluation of prototype

Six expert music therapists with long experience in dementia care took part in four one-day workshops where they were introduced to a prototype of the PAMI training manual. As part of this, they taught each other in the group, and participated in focus group interviews that were recorded and transcribed. Their evaluation of the prototype was integrated in the final version of the PAMI training manual (55).

### Implementation

Intervention implementation is about adapting and transferring theory to practice and practice to theory. For an intervention to be successfully implemented, sustainability in the real-world context is necessary. The implementation of interventions in dementia care should be based on sufficiently testing for feasibility and acceptability, and according to Skivington et al. (42), implementation questions should be considered alongside evaluation questions from the outset.

#### Subproject 11. PAMI training manual

All subprojects added material to the final PAMI training manual, either to the content, to the organization and structure, or to learning objectives and didactics. In the results section, we will present the details of the manual. Throughout the process of constructing the manual, core elements were considered to assure that, for example, the structure was realistic for the learning process and that theory and exercises were meaningful.

#### Subproject 12. PAMI training

The implementation of the PAMI training manual started in September 2021 with a training course for six music therapists for two times 2 days. In April 2023, 19 experienced music therapists working in care homes in different parts of Denmark had completed PAMI-training. Trained music therapists were certified once their report was approved. The report documented the training process of a group of carers following the guidelines of the PAMI-manual. A list of certified music therapists is available at the website www.pami.aau.dk, indicating the ongoing training of not only music therapists, but also of carers.

### Core elements

Throughout the research process there were numerous matters to consider and continually revisit, with each phase connected to the following core elements that are relevant in general for complex intervention research; context, developing and refining programme theory, engaging stakeholders, identifying key uncertainties, refining the intervention, and economic considerations. In addition, ethical considerations were important for us to relate to throughout the process. We continuously needed to consider whether musical interactions are meaningful to the residential care home context and discussed many questions related to how to understand dementia and personhood, the caring culture, caring values, and learning objectives for carers. For example, we considered the following dilemmas and questions: Will it be realistic to ask carers to work with their voice and to hum and sing? How can we best present theory about sensorimotor perception or neurocognition so it is clear and understandable? What is the best way to engage managers in the training course? How do we engage carers and keep them motivated to learn new ways of interaction and to focus on personal awareness? How do we include the perspective of the person with dementia? What is the role of the individual person with dementia in a care home system, but also of relatives? How is it possible to change care home cultures, and even the broader society? How is it possible to balance individual psychosocial needs with structural systems and including needs from relatives? How do we balance the PAMI training course to the economic situation in general for care homes? How do we create a transformative learning culture focusing on non-verbal communication and subjective knowledge? How do we facilitate carers' critical reflexiveness and insight in caring values? As illustrated in Figure 1, these considerations were central to the research process and spurred constant critical reflection.

## Results

The result of the research process was the explication of a theoretical understanding of person-attuned musical interactions, PAMI, as well as the construction of a training manual for music therapists to use when teaching and collaborating with carers about how to implement PAMI in dementia care. During the training, carers work with two tools; the *music care plan* and *lived experience descriptions*. We elaborate on these tools below, but first we describe the combined results of the subprojects.

### Theoretical understanding of PAMI

PAMIs are interactions that we all know from everyday life, but that we seldom use consciously and intentionally. They are termed under the umbrella of non-verbal communication but can be explicated with the use of concepts from the language of music. The key components of PAMI are Person (personhood, personal needs, preferences, identity, caring), Attunement (tuning in, tone of voice, trust, perspective, empathy), Musical (cuing, pitch, tempo, volume, musicking, listening, playing, resonance), and Interaction (moving, sensorimotor integration, communication, mutuality) which are each described in depth in the book Stemning [Attunement] (1).

In care situations where non-verbal communication is crucial, there is a need to describe what happens in a mutual professional language that is agreed upon in the caring culture. Otherwise, what happens easily becomes invisible and with no value. We suggest PAMI as a way to understand caring interactions and to develop the culture of care. PAMIs are used intentionally in music therapy and can be used in caring when carers initially are guided by music therapists. This may help carers to understand their interactions, describe them in the collaborative team, and to implement them as a professional way of interacting in care situations.

Carers do not need to know the full theoretical underpinnings of the PAMI theory, but they do need to understand the basic concepts and to recognize when PAMIs are happening. Such understandings are not learned by reading books, but by experiencing how it is to interact with someone. Therefore, it is important that carers throughout the training relate PAMI to practice and experience how PAMI work in practice. As an example, carers will learn about musical cuing by experiencing it, understanding how it affects the body, and further how to use it as a technique to facilitate safety.

In the understanding of PAMI, it is important to see the main differences of why music is used. The impact of music can be very strong, and if used in the wrong way, it will influence the person with dementia negatively. Carers are therefore instructed to distinguish between Framing, Regulating, and Relating which is elaborated upon throughout the training course in both theory, action learning and counseling. Table 1 gives an overview of the main differences between these uses of music.

**Table 1:** Music has three overall functions in PAMI (Framing, Regulating, Relating) with the overall purpose to provide safety, balance arousal, and meet psychological needs.

	**Music**	**Musical interaction**	**Purpose**
Framing	Selected background music. Songs to start/end. Music-routines. Soundscape	Musical cuing	Safety, predictability
Regulating	Humming, singing. Care-songs. Stimulating/calming music	Arousal regulation with body, voice, music (pitch, volume, tempo)	Balanced arousal. Concentration and contact
Relating	Personal songs. Music to trigger memories and match emotions. Music-reminiscence	Empathic attunement via personal, well-known music and by holding and validating the music experience	Attunement and connectedness. Spontaneous memories. Psychological needs

### Training manual with resources

The training manual is a book of 43 pages with introduction to the idea, content and teaching resources, explaining the structure of the training course, and providing guidelines for the hands-on action learning sessions as well as group and individual counseling. In addition to the training manual, the following resources are provided:

1) PowerPoints for the four teaching modules (for music therapists)
2) Action learning guidelines for the four teaching modules (for music therapists)
3) Booklet (for the participating carers)
4) Theory book (for professionals, managers, and relatives)

By continuously considering core elements for complex intervention research such as learning objectives, economy, and the engagement of stakeholders, we ended up with a training course consisting of four teaching modules, with each module containing 1 hour theory, 1 hour action learning, 1 hour group counseling, and 1 hour direct counseling at the residential care home for each carer. For learning outcomes to be integrated in daily experiences, the 16-hour training course was not intended to be held intensively in just 2 days but should take place as single sessions over a longer period of 8–16 weeks. Each of the four teaching modules involved the overall learning outcomes as listed in Table 2.

**Table 2:** Learning outcomes from the training course in terms of increasing knowledge and developing carers' interaction competencies.

**Module**	**Knowledge**	**Learning outcome: competencies to …**
1. Music and dementia	• Dementia and communication • Body language and voice • Music and singing • The voice as instrument • Research in music and dementia	• Assess own and others' vocal expressions with musical parameters • Assess advantages and disadvantages of using song and music
2. Framing	• Music, brain, and the senses • Hearing and sensing • Perception, perspective, and reality • Safety and predictability • Musical cuing	• Use song and music to create safety and for frame-setting an activity • Use simple care songs for guiding • Use music for activities
3. Regulating	• Cognitive functions • Sensorimotor approach • Arousal regulation • Musical regulation • Care singing	• Assess how arousal, preferences and degree of dementia affect how music is experienced • Use song and music for arousal regulation
4. Relating	• Person-centered care • Psychosocial needs • Empathic attunement • Personal music • Music care plan	• Combine PCC with the use of music • Attune to the person with dementia and adjust communication • Use song in mutual communication • Use music and song to meet psychological needs • Plan, implement, and evaluate interventions as formulated in the music care plan

### Music care plan

The music care plan is a tool to directly implement PAMI in daily care. It provides the carer with suggestions for how to structure initiatives in an easy way and how to follow up with a simple evaluation of the perceived effect. This enables carers to share, develop, and critically evaluate initiatives in the professional team. The music care plan includes information about which, when, and where a care situation is initiated, which musical interaction, and with which function. The assessment of effect consists of a 5-point scale ranging from very negative, negative, non-observable, positive, and very positive and with the possibility to add further comments. A downloadable version of the music care plan is available at www.pami.aau.dk.

### Lived experience descriptions

If meaningful non-verbal caring interactions are not explicated, it is difficult to value them and to evaluate them. Inspired by van Manen's phenomenological approach on subjective descriptions of human experiences (56), the carers are trained to put into words their interactions in a way where they capture the instant moment and what the experience is like. The purpose is to uncover tacit knowledge, emotions, images, body sensations, and associations, and with help from the music therapist, the experiences are transformed to short written narratives. These are used throughout the learning process to facilitate verbalisation and the writing of short texts about PAMI experiences. In each session, lived experience descriptions are developed, with the aim that the carers will continue to describe meaningful non-verbal interactions. The short narrative about Anna and Lis in the introduction is an example of a lived experience description. It is written in third person language, whereas carers are encouraged to write the narrative in first person language. The lived experience descriptions focus on one specific moment, are written in present tense and with details of sensory and emotional experiences in order to connect them as much as possible to practice, although narratives with this quality are different from what is mostly understood as professional language messages.

## Discussion

### Summary

The research group carried out several subprojects, going through four phases of complex intervention research; Developing, Feasibility, Evaluation, and Implementation, continuously considering Core elements related to the carers and caring, the person with dementia, the care home context, structure for training, and learning objectives. The result was a training manual for music therapists to use when teaching and collaborating with carers about how to implement person-attuned musical interactions in dementia care.

### Interpretation

Based on a systematic review and a meta-analysis on the effectiveness of person-centered care on people with dementia, Kim and Park (57) conclude that PCC reduces agitation, neuropsychiatric symptoms, and improves quality of life. They also stress the need of an educational strategy that promotes learning and skill development of carers. In line with this and with the training of carers in PAMI, focusing on caring values, non-verbal communication, and support into how to carry out person-centered care, we expect the PAMI training to lead to changes in the caring culture, such as less behavioral and psychological symptoms of dementia, increased quality of life, and consequently a reduction in, for example, psychotropic medication.

Mohr et al. (58) pointed at the challenges in evaluating the effects of PCC and psychosocial interventions as they consist of multi-component interventions. For example, they recognized music interventions as either arts/creative activities, sensory enhancement, or cognitive training. Therefore, in their systematic review of key intervention categories to provide PCC, Mohr et al. (58) suggest to evaluate “relationship-centered” interventions, as an explicit focus on how to engage in relationships during PCC may yield an added benefit, not just for the person with dementia but also for the carers. Consequently, they recommended the training of carers. In line with this, we expect that PAMI training will lead to a higher level of relational competence and expertise for carers if their communication competencies are developed in a culture where everyday interactions are valued. This may lead to increased job satisfaction and less stress. Therefore, we see an urge to identify core elements of positive, non-verbal interactions between carers and persons with late-stage dementia, and we suggest the training in PAMI as a solution to changing care home cultures. By offering the structure and content for training, we see the need for further research in cooperation with carers, persons with dementia, relatives, music therapists, care home managers, and researchers in psychosocial methods. Apart from understanding the processes, there will also be a need to evaluate the changes in care home cultures due to PAMI-interventions.

In accordance with Bunn et al. (59), we agree that the organizational context will impact on the successful implementation of healthcare initiatives in care homes. They found that a positive culture of care was defined by giving time and resources to staff education and reinforcement of learning, and that feedback on progress encouraged a sense of shared ownership of a given change. A positive culture of care also gave value to being with residents rather than engaging in task-based care. Bunn's framework addresses the gap between implementation theory and practice, and it provides a range of questions to initiate dialogue between researchers, practitioners, and commissioners, which we find useful also in the implementation of PAMI. In this regard, it is clear how much care home cultures vary in all aspects of, for example, organization, expertise, capacity, staffing level, and services. Carers are involved in washing, lifting out of bed, helping with feeding, and in monitoring health, implementing care plans, and maintaining health records. Still, across the OECD, less than half of surveyed countries require that carers hold a license or certification (60).

For PAMI to be successfully implemented, we expect that carers are trained and have worked in dementia care based on the principles of PCC. Thus, the PAMI training demands previous training and competencies. The 16-h inhouse training is designed to be integrated in daily practice which makes it cost effective and less challenging logistically, but it cannot replace the needed primary training of carers. Further, we also expect that the facilitator of the PAMI training is a credentialed music therapist with a least 2 years of work experience in dementia care. PAMI may be viewed as indirect music therapy practice, and its type of skill-sharing is multidimensional which according to McDermott et al. (50) includes not only promoting informed and safe use of music, but also to enable carers to develop self-awareness so they can be attuned to the needs of persons with dementia, promote cross-professional dialogues, and contribute to organizational change (50).

### Limitations

Non-verbal communication is often unconscious and is ambiguous to describe in words. Despite this, we have strived to put the meaning and relevance of musical interactions into words. In this way, we translate interactions to another dimension where they may lose facets of the original practice-imbedded meaning. However, a translation to spoken and written language is needed for developing clinical contexts and disseminating research. Critical thinking in a group is necessary for understanding the reality, and therefore it is crucial to construct a langue for paralinguistic modalities. Still, there is an inborn limitation in describing real life contexts and discussing them at a meta-level of reasoning.

In this study we included the perspectives of carers and of music therapists. As an integrated part of PAMI, the carers worked on how to integrate the perspective of the persons with dementia through the music care plan and the lived experience descriptions. In the research process, we did not include the perspectives of persons with dementia, relatives, and dementia experts, or did a cost-benefit analysis, and we recommend this for future research. In this regard, we find it highly relevant that Franco et al. (61) provided insights into person-centeredness and quality of care by interviewing persons with dementia and their care partners. They found that PCC is essential to the quality of dementia care, and that quality indicators in general over-emphasize technical and disease-specific medical aspects instead of emphasizing quality of care that values the person.

We also want to point out a limitation related to the ideographic nature of the research project, as we constructed PAMI and developed the training manual in a few local care home cultures. Therefore, we cannot generalize from our findings, and see a need for translation and adaptation to a broader cultural reality where, for example, caring values, workplace structure, stakeholder involvement, and economy are different.

### Perspectives

The PAMI core group research took place from 2016 to 2022, with a related project in the UK finalizing in 2023. In Denmark, PAMI training of both music therapists and carers is continuing which enables collection of data from the implementation of PAMI and evaluation of the process and quality on a wider scale. Further implementation, evaluation and piloting will give ground for protocol registration for future RCTs. With a thoroughly developed and tested training manual where its preliminary usefulness has been examined, the initial ideographic approach will feed into a nomothetic design. Relevant outcomes to measure would be the prescription of psychotropic medication, behavioral and psychological symptoms, and quality of life for persons with dementia, as well as job satisfaction and stress level for carers.

## Conclusions

A major part of our non-verbal communication components can be explained in musical terms: such as the tone of voice, the rhythm in speech, and accompanying gestures. Meaningful interactions between people happen when we make attuned relational connection with each other. For persons with late-stage dementia, non-verbal communication is crucial for person-centered care to succeed in meeting their psychological needs. Music therapists have clinical insights into how successful Person-Attuned Musical Interactions can work in dementia care and can train carers in an intentional and systemic use of PAMI. Based on a complex intervention study model, we developed, explored, evaluated, and implemented a PAMI training manual. The PAMI manual includes comprehensive resources, a clear structure for training, defined learning objectives, and integration of theory. With increased knowledge about caring values and non-verbal communication—here in the form of a fully developed PAMI training course—residential care home cultures are offered possibilities to develop carer competencies and to provide professional attuned care for persons with late-stage dementia.

## Data availability statement

Participants identifiable data (audio and video recordings, transcripts) are only available to data managers and are deleted after completion of each of the studies. Datasets are not available, but resources from the research are available at www.pami.aau.dk.

## Ethics statement

The study was granted exemption from the Danish Regional Ethics Committee (Den Videnskabsetiske Komité for Region Nordjylland, January 25, 2016, Project N-20160002) and was registered at the Danish Data Protection Agency through Aalborg University. The study adheres to the Danish Code of Conduct for Research Integrity, Ministry of Higher Education and Science, 2014, and participants signed an agreement on the terms of participating in the study based on written and oral information provided by the researcher.

## Author contributions

HR contributed to conception and design of the study together with OM, JA-I, and JK, and all contributed to selected subprojects. HR wrote the manuscript draft. All authors contributed to manuscript revision, read, and approved the submitted version.

## Footnotes

^1^In our description of the state of art, we have included literature on both nurses and formal and informal caregivers. In the following, we focus on professional residential care home staff, and use the wording *carers*.
